# Long-term continuous corrosion of 316L stainless steel by *Streptococcus mutans* in simulated oral environment

**DOI:** 10.3389/fbioe.2025.1725414

**Published:** 2025-12-05

**Authors:** Xiaodong Zhang, Yao Liu, Guoxian Chen, Shuai Bai, Zhong Li, Fuhui Wang, Dake Xu

**Affiliations:** 1 State Key Laboratory of Digital Steel, School of Materials Science and Engineering, Northeastern University, Northeastern University, Shenyang, China; 2 Key Laboratory for Anisotropy and Texture of Materials (Ministry of Education), School of Materials Science and Engineering, Northeastern University, Shenyang, China

**Keywords:** oral environment, long-term corrosion, microbiologically influenced corrosion, *Streptococcus* mutans, 316L stainless steel

## Abstract

**Introduction:**

The widespread application of metallic biomaterials in oral healthcare has raised increasing concerns regarding their long-term corrosion behavior under complex microbial environments, which remains insufficiently understood.

**Methods:**

A 90-day anaerobic experimental system was established by inoculating *Streptococcus mutans* in artificial saliva. The biofilm formation, electrochemical properties, surface corrosion morphology, and product evolution on 316L stainless steel were monitored using SEM, CLSM, electrochemical analysis (OCP, LPR, EIS), AFM, and XPS.

**Results:**

*S. mutans* formed a dense biofilm on the material surface with a maximum thickness of 88.1 ± 9.0 μm. Compared to the sterile control, the corrosion current density in the experimental group increased by approximately 295-fold, with a maximum pit depth of 5.3 μm. A notable reduction in the content of protective Cr_2_O_3_ and NiO within the passive film was observed.

**Discussion:**

*S. mutans* accelerates pitting corrosion through a “biofilm barrier + localized acid production” mechanism that disrupts the passive film. The long-term corrosion effects are substantially more severe than those observed in short-term experiments.

## 1 Introduction

The oral cavity, as the initial site of food digestion, plays a pivotal role in maintaining systemic health. According to the World Health Organization Global Oral Health Status Report, approximately 3.5 billion people worldwide are affected by oral diseases (Eduok, 2024). In China alone, the prevalence of dental caries among 5-year-old children reached 66% (Zou et al., 2018). In response to the growing burden of oral health issues over recent decades, a wide array of metal-based materials, including various alloys, have been extensively employed in orthodontic wires, restorations, and dental implants (Sheit et al., 2024). The oral environment is inherently complex, comprising water, proteins, enzymes, and electrolytes, all of which can profoundly influence the stability and performance of metal biomaterials (Gopalakrishnan et al., 2021). Furthermore, its warm and moist nature creates a favorable niche for microbial colonization, placing dental implants in prolonged contact with diverse oral microorganisms. It is estimated that the oral microbiome comprises over 700 species, including bacteria, fungi, archaea, viruses, and protozoa (Dzidic et al., 2018; Lee et al., 2021; Perera et al., 2022). A growing body of evidence suggests that many oral microbes can accelerate the deterioration of metallic biomaterials through microbiologically influenced corrosion (MIC), leading to surface degradation, compromised mechanical integrity, and significantly reduced service life of dental devices (Xu et al., 2024). Notably, up to 24% of implant failures have been directly linked to corrosion-related issues (Asri et al., 2017; Lodhi et al., 2019). Metal corrosion can also result in the release of toxic metal ions (Gopalakrishnan et al., 2021), which may induce adverse physiological responses such as tissue inflammation, infection, allergic reactions, and even more severe health complications (Olmedo et al., 2013; Xu et al., 2018; Eduok, 2024). Therefore, a mechanistic understanding of MIC in oral metallic materials is essential for assessing their long-term performance and developing effective strategies for MIC mitigation.

*Streptococcus mutans*, a Gram-positive facultative anaerobe and a dominant resident of the oral cavity, is characterized by its strong acidogenicity, high aciduricity, and robust biofilm-forming ability (Kaur et al., 2015; Madiba et al., 2023). As one of the principal etiological agents of dental caries, *Streptococcus mutans* not only contributes to enamel demineralization but also accelerates the corrosion of oral metallic materials due to its acid production (Wong et al., 2017; Alam et al., 2018; Lemos et al., 2019; Yang Y. et al., 2021). *S. mutans* can efficiently metabolize sucrose to synthesize extracellular polysaccharides, which serve as essential structural components of biofilms and facilitate stable bacterial adhesion to surfaces (Bowen and Koo, 2011). The dense and adhesive biofilm formed on metal surfaces significantly modulates the progression and kinetics of MIC (Gu et al., 2019; Jia et al., 2019). Notably, *S. mutans* has been reported to markedly enhance the corrosion rates of various dental alloys, such as 316L stainless steel (SS), 304 SS, and Ti6Al4V alloy, etc. (Kameda et al., 2014; Yang C. et al., 2021; Zhou et al., 2023).

Biofilms represent a distinct microbial lifestyle that enhances microbial competitiveness and resistance to harsh environmental conditions (Hong et al., 2021; Tuck et al., 2022), and they are recognized as a critical factor in the initiation and progression of MIC (Blackwood, 2018; Procopio, 2019; Li et al., 2023). As a dynamic and evolving structure, the composition, architecture, and physicochemical properties of biofilms vary significantly over time, thereby introducing considerable complexity to their impact on material corrosion (Li and Ning, 2019). Microbial activity within biofilms alters the physicochemical conditions at the biofilm–material interface, including dissolved oxygen levels, chloride and other ion concentrations, and local pH, all of which critically influence corrosion kinetics (Madirisha et al., 2022). Recent studies have also revealed that electroactive microbes can directly extract electrons from metallic surfaces, thereby accelerating corrosion through a mechanism known as extracellular electron transfer MIC (EET-MIC). Furthermore, the inherent diffusional resistance of biofilms has been shown to create significant pH gradients between the regions beneath the biofilm and the bulk solution (Fulaz et al., 2019; Hollmann et al., 2021). Such physicochemical heterogeneity, coupled with the uneven distribution of biofilm constituents, may lead to the formation of localized galvanic cells, thereby promoting corrosion initiation and propagation (Xu et al., 2024). Additionally, extracellular polymeric substances (EPS) within the biofilm matrix contain anionic functional groups capable of chelating metal cations, which can also induce metal dissolution or alter material properties (Beech and Sunner, 2004).

In modern biomedical applications, 316L SS has been extensively employed as a dental material due to its excellent biocompatibility, outstanding corrosion resistance, favorable mechanical properties, and relatively low manufacturing cost (Tang et al., 2006; Hayes et al., 2015; Liu et al., 2025). The superior corrosion resistance of 316L SS is primarily attributed to the spontaneous formation of a dense chromium-rich passive film on its surface. However, in the oral environment, microbial activity can disrupt this protective layer, compromising its barrier function against aggressive species. This disruption often leads to localized breakdown, such as pitting corrosion, and promotes the release of metal ions (Wang et al., 2022; Zheng et al., 2024). Microbial acid production and EET processes have been identified as key contributors to the corrosion of 316L SS.

Although several studies have explored MIC in oral environments, most investigations have been limited to short-term exposure periods, typically ranging from 7 to 14 days (Wang et al., 2022; Zheng et al., 2024; Zhou et al., 2025). Such short timeline fails to capture the complex and evolving conditions experienced by implants during prolonged clinical service. In reality, the corrosion process is governed by multifactorial interactions involving microbial metabolism, biofilm development, and dynamic electrochemical environments (Xu et al., 2023). With prolonged service time, the physicochemical environment beneath the biofilm undergoes significant changes, thereby influencing the corrosion process. Consequently, to accurately elucidate the long-term effects of oral microbes on biomedical metals, extended-duration evaluations are of great importance.

Despite the widespread clinical application of SS in the oral cavity (Oh et al., 2004), a comprehensive understanding of its corrosion behavior and underlying mechanisms under prolonged microbial exposure remains limited. Herein, an *in vitro* anaerobic oral corrosion system was developed to elucidate the long-term (90 days) impact of *S*. *mutans* biofilm on the corrosion of 316L SS. A combination of continuous biofilm characterization, electrochemical measurements, pH monitoring, surface potential mapping, corrosion morphology analysis, and characterization of corrosion products was employed to elucidate the evolution of MIC process. By moving beyond the constraints of short-term simulations, this work aims to provide experimental evidence toward assessing the long-term corrosion behavior of 316L SS in simulated oral environment.

## 2 Materials and methods

### 2.1 Materials

The 316L SS used in this study had the following chemical composition (wt%): C 0.019–0.024, Mn 1.18, P 0.032, S 0.0006, Si 0.43, Ni 10.5, Cr 16.78, Mo 2.09, and balanced Fe. The material was wire cut into square specimens (10 mm × 10 mm × 5 mm). Prior to use, the samples were sequentially polished using silicon carbide (SiC) papers with varying grit sizes (240, 400, 600, 800, and 1000-grit).

Before conducting immersion tests, the samples underwent ultrasonic cleaning in absolute ethanol for 15 min, followed by air-drying with a cold air stream and UV sterilization in a biosafety cabinet for 20 min. For X-ray photoelectron spectroscopy (XPS) analysis, specimens with sizes of 5 mm × 5 mm × 2 mm were prepared, following the same surface treatment and sterilization protocol as for immersion tests.

### 2.2 Bacterial cultivation

The *S. mutans* strain (ATCC 25175) was used in this study. To ensure microbial viability, the strain was initially resuscitated in Brain Heart Infusion (BHI) broth at 37 °C for 24 h, and then transferred at 1% (v/v) into fresh BHI broth for an additional 6 h. The resulting culture was then used for subsequent immersion and electrochemical tests. *S*. *mutans* was cultivated under aerobic conditions for the resuscitation and activation processes.

Both immersion and electrochemical tests were conducted using an artificial saliva medium with the following components: sucrose (5 g/L), acid-hydrolyzed casein (5 g/L), NaCl (125.6 mg/L), KCl (963.9 mg/L), KH_2_PO_4_ (654.5 mg/L), Na_2_SO_4_ (336.6 mg/L), NH_4_Cl (177.8 mg/L), urea (200 mg/L), NaHCO_3_ (630.8 mg/L), and CaCl_2_ (191.1 mg/L). During preparation, the solution containing NaCl, KCl, KH_2_PO_4_, Na_2_SO_4_, NH_4_Cl, sucrose, and acid-hydrolyzed casein was autoclaved at 115 °C for 30 min. After cooling, sterile-filtered urea, NaHCO_3_, and CaCl_2_ were supplemented sequentially. The pH was then adjusted to 7.4 ± 0.1 using 5% (v/v) phosphoric acid. Prior to use, the artificial saliva medium was deoxygenated by purging with high-purity N_2_ gas passed through a 0.22 μm filter. All corrosion tests were performed under strict nitrogen protection, and sterile controls were included in parallel.

### 2.3 Long-term immersion test

To examine the evolution of biofilm formation, corrosion products, and surface morphology during long-term MIC tests, 316L SS samples were immersed in 50 mL anaerobic serum bottles containing 30 mL of artificial saliva medium. The medium was inoculated with *S. mutans* at 1% (v/v) or not. The anaerobic bottles were sealed with butyl rubber stoppers and aluminum crimps to ensure a strictly anaerobic environment, and then incubated statically at 37 °C. To provide a continuous nutrient supply for *S. mutans* throughout the 90-day experimental period, the culture medium was replaced every 15 days. All medium exchanges were conducted under anaerobic conditions using a sterile syringe to carefully withdraw the spent medium, which was then replaced with fresh, sterile, and deoxygenated medium. After each medium replacement, 1% (v/v) fresh *S. mutans* suspension was also added to ensure microbial viability. All equipment used for the immersion tests was sterilized by autoclaving at 121 °C for 20 min to prevent microbial contaminants.

### 2.4 Long-term electrochemical testing setup

Long-term electrochemical testing was conducted in a custom-designed 5-L glass bioreactor (BLBIO-5GJ, Bailun Bio, China). The bioreactor chamber was made of borosilicate glass and sealed with polytetrafluoroethylene (PTFE) ports to ensure gas-tightness and biocompatibility. The detailed design is described in a Chinese Patent (No. ZL202020179911.7). The reactor included dedicated inlet and outlet ports for medium addition and replacement, as well as gas inlet and outlet ports for purging with high-purity nitrogen (filtered through a 0.22 μm membrane) to achieve deoxygenation. All components were sterilized by autoclaving after assembly, and all operations involving medium handling were performed under strict aseptic conditions. To maintain the sustained viability of *S. mutans* throughout the 90-day experimental period, the culture medium was replaced with fresh artificial saliva medium at 15-day intervals. After medium exchange via the inlet and outlet ports, fresh *S. mutans* broth was also added at 1% (v/v) relative to the total medium volume. The reactor was then sealed and purged with high-purity nitrogen for 1 h to remove residual oxygen.

### 2.5 Electrochemical corrosion testing

Electrochemical measurements were performed utilizing a three-electrode cell connected with an electrochemical workstation. (Reference 600, Gamry Instruments, USA). The three-electrode cell comprised a saturated calomel electrode serving as the reference electrode, a platinum electrode acting as the counter electrode, and a 316L SS working electrode with an exposed surface area of 1 cm^2^. The 316L SS working electrode was prepared as follows: the sample surface was initially polished using 240-grit SiC paper, then embedded in polyester thermosetting powder and cured at 180 °C. After mounting, both sides of the sample were further polished with 240-grit SiC paper to fully expose the metal surface. A 0.5 mm diameter copper wire was attached to the back of the sample using conductive adhesive. The electrode was then mounted in epoxy resin. Prior to electrochemical testing, the working surface was sequentially polished using SiC papers with varying grit sizes (240, 400, 600, 800, and 1000-grit) to ensure surface consistency.

Electrochemical tests were conducted using the long-term immersion setup described above containing 1 L of simulated saliva medium, with or without *S. mutans* inoculation. Open circuit potential (OCP), linear polarization resistance (LPR), electrochemical impedance spectroscopy (EIS), and potentiodynamic polarization tests were performed. After OCP was stabilized, subsequent electrochemical measurements were carried out. LPR measurements were conducted at a scan rate of 0.125 mV/s within a potential range of −10 to +10 mV vs. *E*_OCP_. EIS data were recorded over a frequency range of 100 kHz to 0.01 Hz with a sinusoidal perturbation amplitude of 5 mV, and analyzed using ZSimpWin software (version 3.60, Princeton Applied Research, USA). Potentiodynamic polarization measurements were conducted on day 90, with the potential scanning from −0.3 V to +1.5 V vs. *E*_OCP_ at a constant scan rate of 0.333 mV/s. Corrosion potential (*E*_corr_) and corrosion current density (*i*_corr_) were determined by Tafel extrapolation of the cathodic branch using Gamry Echem Analyst software (version 7.10.3, Gamry Instruments, USA). OCP and LPR measurements were performed on days 0, 7, 15, 22, 30, 37, 45, 52, 60, 67, 75, 82, and 90, while EIS experiment was conducted on days 3, 7, 15, 30, 60, and 90. All electrochemical measurements were performed prior to the medium replacement.

### 2.6 Analysis of corrosion morphology on 316L SS surfaces

After 90 days of immersion in either sterile or *S. mutans*-inoculated media, 316L SS samples were immersed in Clark’s solution for 10 s according to ASTM G1–03 (Li et al., 2024) to remove corrosion products and biofilms, then ultrasonicated in absolute ethanol for 30 min and air-dried. The corrosion morphology was characterized using confocal laser scanning microscopy (CLSM, LSM 900, Zeiss, Germany) in reflection mode to visualize the micromorphology of corrosion pits. For statistical analysis, at least 10 randomly selected fields were examined per condition to measure the width and depth of corrosion pits, and to determine the average, maximum, and distribution of corrosion pits.

### 2.7 Stochastic model for the probability of pitting corrosion occurrence

In this work, a stochastic model was used to quantify the probability of pitting corrosion occurrence. The cumulative probability *F(Y)* was calculated using Equation 1, wherein *N* represents the total number of data points and *n* denotes the rank of each pitting corrosion value in descending order. The variable *Y* was defined by Equation 2:

$$F\left(Y\right)=1-\frac{n}{N+1}$$ (1)

$$Y=-\ln\left\{-\ln\left[F\left(Y\right)\right]\right\}$$ (2)

The probability of pit size occurrence was predicted using the Gumbel extreme value distribution, with μ and α calculated from Equation 3 and incorporated into the double exponential function of Equation 4.

$${\text{Pit}}_{\max}=\mu +\alpha \ln S$$ (3)

$$P=1-\exp\left\{-\exp\left[\frac{-\left(d_{i}-\left[\mu +\alpha \ln S\right]\right)}{\alpha }\right]\right\}$$ (4)

where *d*_*i*_ represents the pit depth, *μ* is the location parameter (i.e., the most probable value), *α* is the scale parameter indicating the spread of the data point distribution, and *S* is the total surface area of the coupon (*S* = 1/cm^2^) (Jin et al., 2024).

### 2.8 Analysis of *S. mutans* biofilm

After immersion in *S. mutans*-inoculated medium for 3, 7, 15, 30, 60, and 90 days, the 316L SS samples were carefully collected to observe the micromorphology of the biofilms using a scanning electron microscopy (SEM, EVO 10, Zeiss, Germany). At each time point, samples were gently rinsed with phosphate-buffered saline (PBS) to remove planktonic bacteria, then fixed in 4% (v/v) glutaraldehyde at 4 °C for 4 h. Dehydration was carried out through immersion in graded concentrations of ethanol (50%, 60%, 70%, 80%, 90%, 95%, and 100% v/v, 6 min each). Prior to SEM observation, samples were sputter-coated with a layer of gold to enhance conductivity.

Biofilms on 316L SS surfaces were further characterized by CLSM to assess their three-dimensional (3D) structure, thickness, surface coverage, and the spatial distribution of live and dead cells. Samples were collected at days 3, 7, 15, 30, 60, and 90, gently rinsed with PBS, and stained in the dark for 15 min using a mixture of 75 nM SYTO-9 and 450 nM propidium iodide (Live/Dead BacLight™ Bacterial Viability Kit, L7012, Life Technologies, USA). After staining, samples were air-dried and examined by CLSM. Live cells emitted green fluorescence under 488 nm excitation, while dead cells showed red fluorescence under 559 nm excitation.

### 2.9 pH measurement

During the 90-day of MIC test, 3 mL of the immersion medium was periodically withdrawn under anaerobic conditions for pH measurement using a calibrated pH meter (FE28-Standard, Mettler Toledo, United States). Sampling was conducted on days 0, 7, 15, 22, 30, 37, 45, 52, 60, 67, 75, 82, and 90, in parallel with electrochemical measurements of OCP and LPR. Particular attention was paid to pH variations before and after medium replacement. All experiments were performed in triplicate under identical conditions to ensure reproducibility and data reliability.

### 2.10 Characterization of corrosion products

After incubation for 90 days in sterile saliva medium and *S. mutans* broth, the 316L SS samples were retrieved, rinsed in PBS to remove biofilms and planktonic bacteria, and then air-dried under a cold stream. XPS (ESCALAB250 surface analysis system, Thermo VG, USA) was utilized to analyze the chemical composition of corrosion products on the surfaces of 316L SS. A wide-scan survey was performed over a binding energy range of 0–1,400 eV. High-resolution spectra were acquired using a monochromatic Al Kα X-ray source (photon energy 1,500 eV, power 150 W) with a step size of 0.2 eV within a 50 eV range. The binding energies of each XPS spectrum were calibrated using the C 1s peak at 284.8 eV. Calibration and fitting of peaks were conducted using the Avantage software (version 6.6.0, Thermo Fisher Scientific, USA).

### 2.11 Analysis of surface potential

For surface potential analysis, 316L SS samples were finely polished using a series of SiC papers (1,000–5,000 grit), followed by 0.5 μm diamond paste and 50 nm colloidal silica to obtain a mirror finish. After immersion for 90 days in either sterile saliva medium or *S. mutans* inoculated media, samples were cleaned with ethanol and sonicated for 30 min to remove biofilms. Surface potential mapping was conducted under ambient conditions using the scanning Kelvin probe force microscopy (SKPFM) mode of an atomic force microscopy (AFM, Cypher ES, Oxford Instruments, USA). The AFM probes were coated with a Cr/Co alloy (HQ:NSC18/Co-Cr/Al BS, MikroMasch, Estonia), with a resonance frequency of ∼73.13 kHz and a calibrated spring constant of 2.12 N/m. The scanning speed was 1.55 Hz.

## 3 Results

### 3.1 Dynamic evolution of microbial biofilms

**FIGURE 1:**
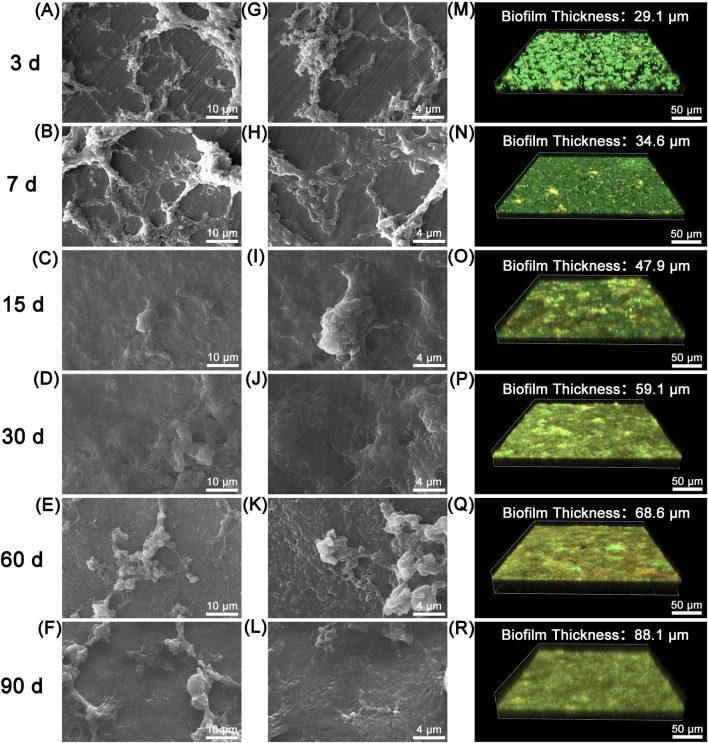
SEM and CLSM images of *S. mutans* biofilms formed on 316L SS surfaces at different time points: **(A–F)** and **(G–L)** show SEM images of biofilms at 3, 7, 15, 30, 60, and 90 days, respectively; **(M–R)** present the corresponding 3D-CLSM images at each time point.

The formation of microbial biofilms is a key factor in the accelerated corrosion of metallic materials (Rao and Mulky, 2023). To investigate the colonization and development of *S. mutans* biofilm on the surface of 316L SS, we employed SEM and CLSM to characterize the morphology and viability (live/dead status) of biofilms during the 90-day of immersion in a simulated oral environment. SEM images revealed the progressive colonization and thickening of *S. mutans* biofilms on the surface of 316L SS (Figure 1). After 3 days of incubation, the biofilms were unevenly distributed across the metal surface, with clustered microbial aggregates visible, while some regions of the bare metal remained exposed. At this stage, the characteristic chain-like morphology derived from division of *S. mutans* can be clearly detected. After 15 days, the surface morphology underwent a dramatic transformation. The metallic matrix was no longer visible, nor were the chain-like bacterial structures. In contrast, a dense and continuous biofilm fully covered the metal surface. This compact biofilm layer persisted through to 90 days. Notably, after 60 days, clustered structures became increasingly evident on the biofilm surface. These may represent mature biofilm architectures, through which adherent bacteria detach and disperse into the surrounding medium.

The spatial structure, thickness, and viability of the biofilms were further characterized using CLSM combined with live/dead fluorescence staining. The CLSM images clearly indicated the transition of the biofilm from a loose to a compact structure over time, consistent with the SEM observations. From day 3 to day 15, the biofilm gradually became denser (Figures 1M–O). On days 3 and 7, the biofilms appeared predominantly green, indicating that the majority of *S. mutans* cells were viable (Figures 1M,N). Only a few yellow regions were observed, representing an overlap of green and red fluorescence, suggesting the presence of localized cell death. After 15 days, the proportion of dead cells increased and became more evenly distributed throughout the biofilm. This may be attributed to nutrient depletion and the accumulation of metabolic byproducts within the biofilm matrix.

**FIGURE 2:**
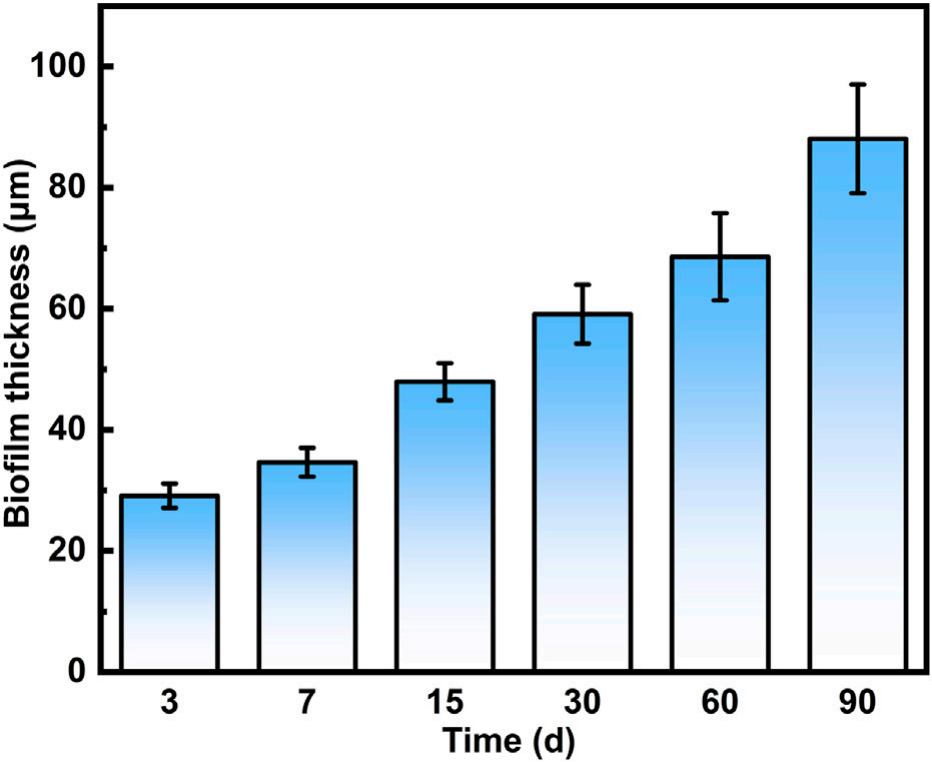
Thickness of *S. mutans* biofilms formed on 316L SS at different time points.

In addition, the average biofilm thickness on the 316L SS surface at each time point was analyzed based on 3D-CLSM images (Figure 2). A progressive increase in biofilm thickness was detected with prolonged incubation. By day 90, the biofilm reached a maximum thickness of 88.1 ± 9.0 μm. These findings highlight the strong biofilm-forming ability of *S. mutans*. The formation of such a dense biofilm can act as a physical barrier, limiting mass transport and creating a highly localized and aggressive microenvironment, thereby altering the interfacial electrochemical behavior of the underlying metal (Moradi et al., 2022).

### 3.2 Electrochemical corrosion tests

**FIGURE 3:**
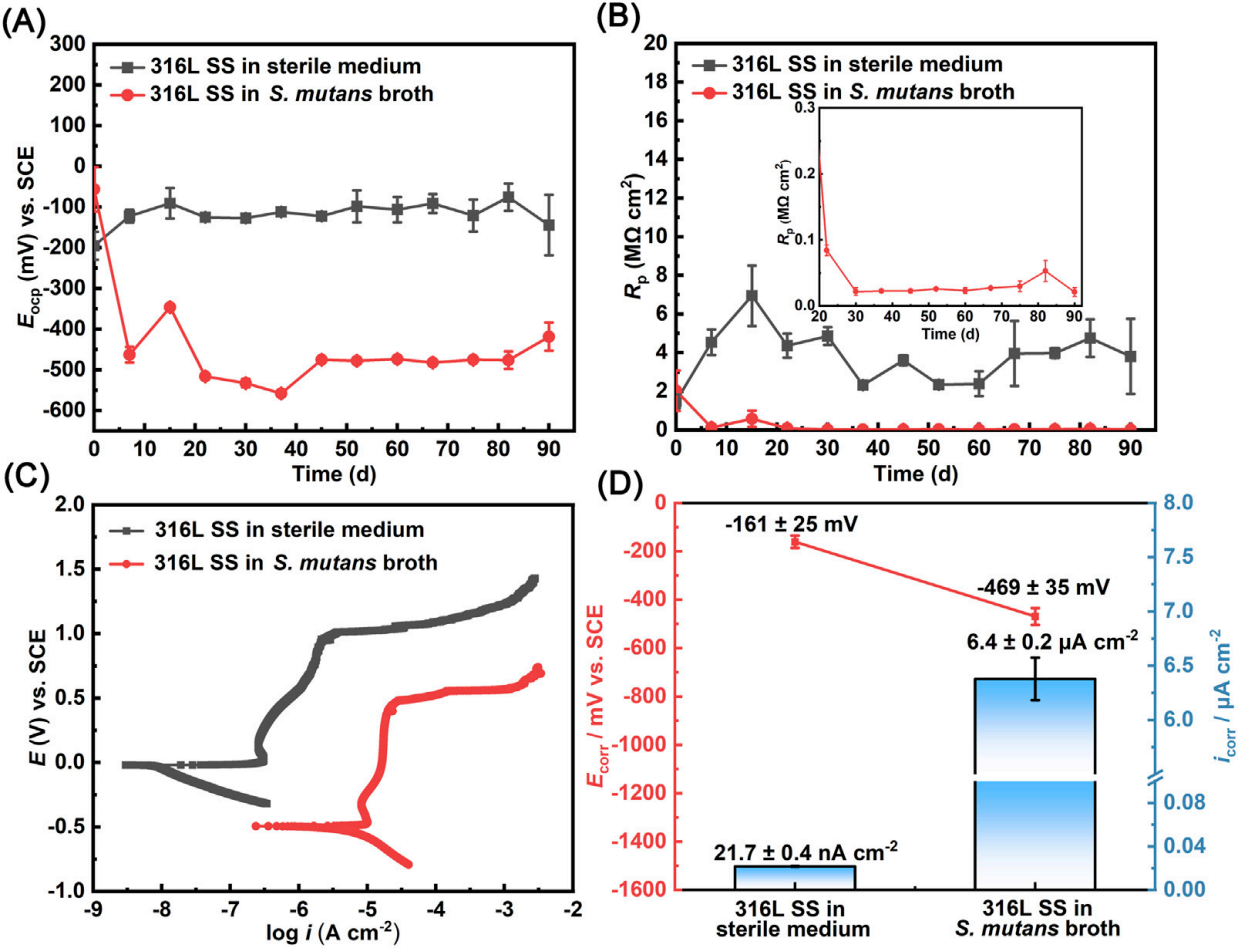
Electrochemical tests of 316L SS in sterile and *S. mutans*-inoculated media: **(A)** variation of *E*_OCP_ over 90 days, **(B)** *R*_p_ value, **(C)** potentiodynamic polarization curves on day 90, and **(D)** *E*_corr_ and *i*_corr_ derived from Tafel extrapolation.

A series of electrochemical measurements were conducted over a 90-day period using a Long-term electrochemical testing setup. The OCP indicates the thermodynamic tendency for corrosion at the material–electrolyte interface and is widely used as a key parameter to assess electrochemical stability (Chiniforoush et al., 2025). As shown in Figure 3A, under sterile conditions, the *E*_OCP_ of 316L SS exhibited an increasing trend during the initial 15 days, rising from −195 ± 34 mV to −91 ± 37 mV. This upward shift is attributed to the spontaneous passivation on the metal surface, which progressively reduced the anodic reaction rate and increased the potential. Once the passive layer stabilized, the *E*_OCP_ values during the subsequent period fluctuated within a narrow range around −112 ± 20 mV, indicating a relatively stable condition of the metal surface. In contrast, for samples immersed in the *S. mutans* broth, the *E*_OCP_ of 316L SS declined rapidly after immersion. Within 7 days, the *E*_OCP_ dropped from an initial value of −57 ± 54 mV to −462 ± 19 mV, and further decreased to −558 ± 3 mV by day 37. This abrupt shift should be primarily caused by microbial metabolic activity, which significantly altered the interfacial electrochemical conditions and increased the thermodynamic corrosion tendency. Such behavior indicates an elevated risk of localized corrosion. After day 45, the *E*_OCP_ remained relatively stable at approximately −475 mV, suggesting a new equilibrium state at the biofilm–metal interface.

Polarization resistance (*R*_p_) is a key parameter for evaluating the instantaneous corrosion rate of a material (Nishikata et al., 2005), with higher *R*_p_ values indicating lower corrosion rates. As shown in Figure 3B, the variation of *R*_p_ for 316L SS was monitored under both sterile and *S. mutans*-inoculated conditions. Under sterile conditions, *R*_p_ increased rapidly during the first 15 days, rising from an initial value of 1.5 ± 0.4 MΩ cm^2^ to 6.9 ± 1.6 MΩ cm^2^. This trend was consistent with the *E*_OCP_ results and should be attributed to the formation and growth of passive film on the metal surface. In the subsequent testing period, the *R*_p_ values under sterile conditions exhibited some fluctuations but remained with an average value of 3.6 ± 1.2 MΩ cm^2^. This further demonstrates the excellent corrosion resistance of 316L SS, as the passive film remained stable in the absence of microbial activity. The passive film effectively acted as a barrier, preventing aggressive ions such as Cl^−^ and PO_4_^3‒^ in the artificial saliva from penetrating and attacking the metal substrate.

In contrast, the presence of *S. mutans* significantly reduced the *R*_p_ values of 316L SS. As shown, *R*_p_ values dropped sharply from 2.0 ± 1.1 MΩ cm^2^ to 29.6 ± 8.1 kΩ cm^2^ within the first 7 days, confirming that microbial activity markedly accelerated the corrosion process of 316L SS. As the immersion period extended to 90 days, *R*_p_ remained at consistently low levels, reaching 20.9 ± 6.6 kΩ cm^2^ on day 90. This pronounced decline can be attributed to the degradation of the passive film integrity, which significantly reduced the charge transfer resistance and consequently weakened the corrosion resistance of the material.

Potentiodynamic polarization tests conducted on day 90 revealed that the presence of *S. mutans* significantly altered the polarization behavior of 316L SS (Figure 3C). Compared with the sterile control, the polarization curve in *S. mutans*-inoculated conditions shifted markedly toward right, indicating the accelerated corrosion tendency caused by microbial activity. Notably, the pitting breakdown potential (*E*_pit_) of the sample in the *S. mutans* broth decreased significantly from 1.01 V (sterile control) to 0.45 V, a negative shift of 0.56 V. This suggests that the microbial activity destabilized the passive film and enhanced the pitting susceptibility of 316L SS. Tafel extrapolation of the polarization curves was used to determine *E*_corr_ and *i*_corr_ (Figure 3D). In the sterile control group, *E*_corr_ was −161 ± 25 mV and *i*_corr_ was 21.7 ± 0.4 nA cm^−2^. In contrast, in the *S. mutans*-inoculated system, *E*_corr_ shifted negatively to −469 ± 35 mV, and *i*_corr_ increased to 6.4 ± 0.2 μA cm^−2^. These results clearly demonstrate that the long-term exposure to *S. mutans* significantly accelerated the corrosion rate of 316L SS. We further analyzed the anodic (*β*_a_) and cathodic (*β*_c_) Tafel slopes based on the potentiodynamic polarization curves. In sterile medium, the *β*_a_ was determined to be 311 ± 15 mV/dec. However, in the *S. mutans* broth, *β*_a_ decreased significantly to 154 ± 10 mV/dec, indicating an increased tendency toward anodic dissolution due to the presence of the microbial biofilm. A notable increase in *β*_c_ was also observed in the presence of *S. mutans* (Table 1), which is likely attributed to the dense biofilm hindering mass transport. This diffusion limitation may have suppressed the cathodic reaction kinetics, leading to a reduced cathodic reaction tendency.

**TABLE 1:** Fitted Tafel slopes from potentiodynamic polarization curves.

Experimental condition	*β*_a_ (mV/dec)	*β*_c_ (mV/dec)
316L SS in sterile medium	311 ± 15	158 ± 26
316L SS in *S. mutans* broth	154 ± 10	260 ± 42

**FIGURE 4:**
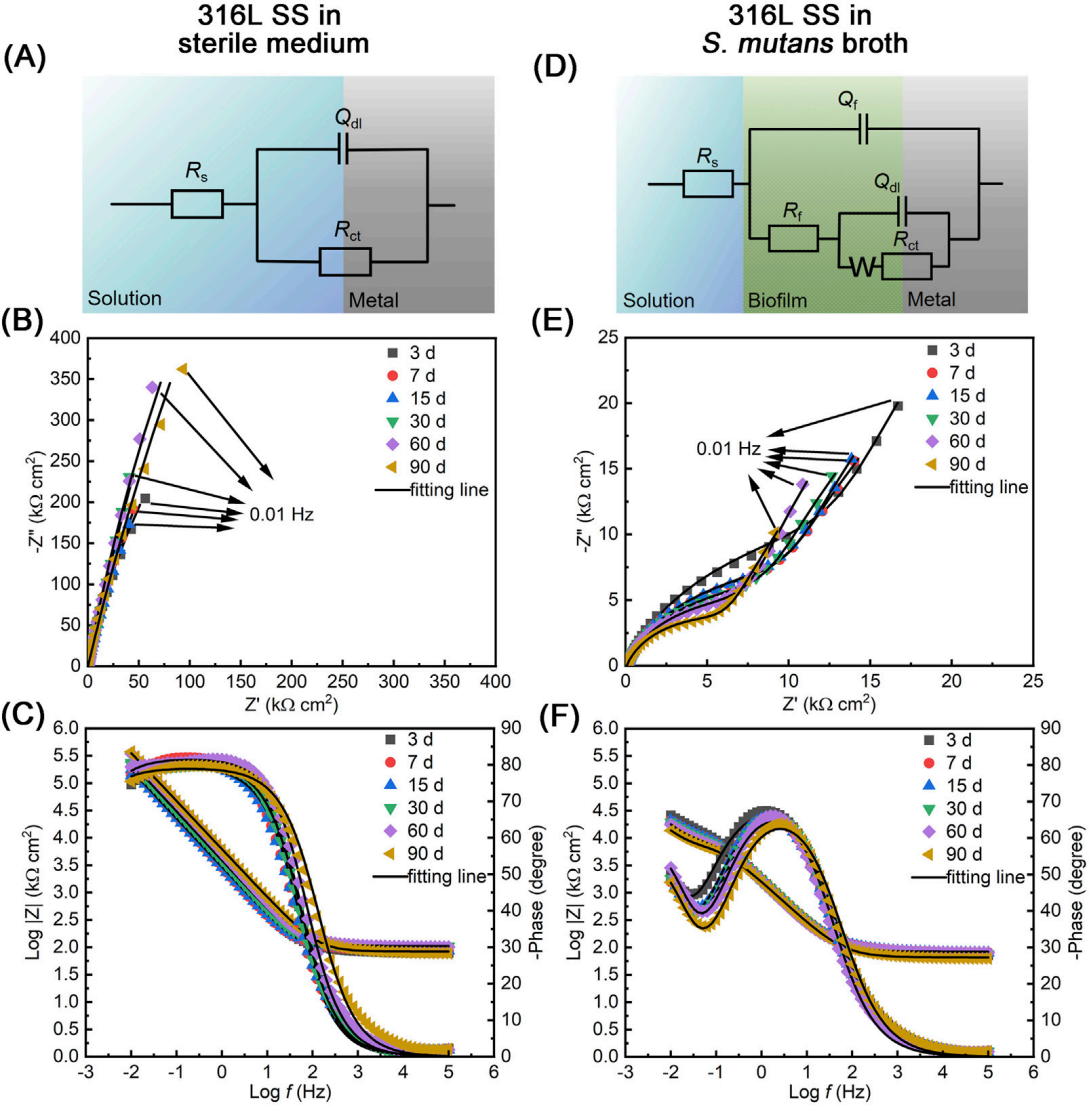
EIS results of 316L SS during the immersion in sterile and *S. mutans*-inoculated media for different time (at 3, 7, 15, 30, 60, and 90 days): **(A,D)** equivalent circuit models used for fitting the EIS data under sterile and bacterial conditions, respectively; **(B,E)** Nyquist plots of 316L SS under sterile and bacterial conditions; **(C,F)** Bode plots under sterile and bacterial conditions. Experimental data points and corresponding fitting curves are included in the plots.

EIS, a nondestructive and highly sensitive electrochemical technique (Manohar et al., 2008), was employed to investigate the interfacial electrochemical reactions of 316L SS in both sterile and *S. mutans*-inoculated media at different time points. The corresponding Nyquist and Bode plots are shown in Figures 4B,C,E,F, respectively. The equivalent electrical circuits used for fitting EIS data are presented in Figures 4A,D, and the associated fitting parameters are summarized in Table 2. The diameter of the capacitive arc in Nyquist plots reflects the combined effects of double-layer capacitance and charge transfer resistance at the metal–electrolyte interface. In the sterile medium, no additional peaks were observed in the phase angle curve of the Bode plot; therefore, the R (QR) model was employed for fitting the EIS data. The arc radius gradually increased over the first 15 days, indicating a significant rise in charge transfer resistance (Figure 4B). Meanwhile, in the Bode plots (Figure 4C), the impedance modulus |Z| in the low-frequency region increased markedly, which is generally associated with improved surface stability and the formation of protective films. These trends collectively indicate the progressive formation of a compact and stable passive film on the 316L SS surface. After day 15, the capacitive arc radius remained nearly constant, suggesting that the passive film effectively suppressed anodic metal dissolution and remained stable throughout the 90-day period.

**TABLE 2:** EIS fitting parameters of 316L SS immersed in different culture media.

Time (d)	*R*_s_ (Ω cm^2^)	*Q*_f_ (Ω^−1^ cm^−2^ s^n^)	*n*_1_	*R*_f_ (Ω cm^2^)	*Q*_d1_ (Ω^−1^ cm^−2^ s^n^)	*n*_2_	*R*_ct_ (Ω cm^2^)	*W*_c_ (Ω cm^2^ s^−0.5^)
316L SS in sterile medium								
3	82.8				5.9 × 10^–5^	0.90	1.9 × 10^6^	
7	90.8				6.4 × 10^–5^	0.91	2.3 × 10^6^	
15	91.5				6.8 × 10^–5^	0.87	5.0 × 10^6^	
30	105.4				5.0 × 10^–5^	0.90	9.7 × 10^6^	
60	105.3				3.5 × 10^–5^	0.91	5.7 × 10^6^	
90	83.5				3.2 × 10^–5^	0.88	8.4 × 10^6^	
316L SS in *S. mutans* broth								
3	75.2	1.3 × 10^–4^	0.79	2.6 × 10^4^	6.2 × 10^–4^	1.00	1.2 × 10^5^	3.3
7	84.5	1.3 × 10^–4^	0.80	1.7 × 10^4^	7.8 × 10^–4^	1.00	5.7 × 10^4^	1.9 × 10^3^
15	83.7	1.3 × 10^–4^	0.80	1.8 × 10^4^	7.6 × 10^–4^	1.00	6.0 × 10^4^	5.0 × 10^3^
30	77.8	1.3 × 10^–4^	0.80	1.5 × 10^4^	8.0 × 10^–4^	1.00	4.2 × 10^4^	5.0 × 10^3^
60	83.4	1.4 × 10^–4^	0.81	1.2 × 10^4^	8.1 × 10^–4^	1.00	3.5 × 10^4^	7.7 × 10^3^
90	65.6	1.4 × 10^–4^	0.79	9.3 × 10^3^	1.1 × 10^–3^	1.00	1.6 × 10^4^	5.9 × 10^3^

Compared with the sterile condition, the impedance resistance of 316L SS showed a pronounced decrease after inoculation with *S. mutans* (Figures 4E,F). The diameter of the capacitive arc in Nyquist plots exhibited a dramatic reduction over the 90-d immersion, indicating a significant decrease in charge transfer resistance and suggesting that electron transfer at the electrode surface became much easier. Simultaneously, the impedance modulus |Z| in the low-frequency region of the Bode plots decreased by approximately two orders of magnitude compared to sterile condition, implying severe degradation of the passive layer on 316L SS surface. In the *S. mutans* broth, the appearance of a linear segment in the low-frequency region indicates a diffusion-controlled electrode process. Consequently, the Warburg impedance was introduced during the fitting process to describe the diffusion-limiting effect of the biofilm on the interfacial reactions. Furthermore, an additional peak was observed in the phase angle curve of the Bode plot, indicating the presence of two dominant interfacial reactions. Therefore, the R (Q (R (Q (RW)))) model was employed for fitting the EIS data. With prolonged incubation, the Warburg impedance exhibited a progressive increase (Table 2), indicative of enhanced diffusion resistance. This trend indicates the densification of the biofilm increasingly impedes mass transport. At day 90, however, a slight decrease in Warburg impedance was observed, likely due to the partial disruption of the biofilm structure caused by the death of sessile bacteria. This loss of structural integrity may have reduced the biofilm’s barrier function, thereby decreasing its overall diffusion resistance.

### 3.3 Characterization of corrosion morphology and corrosion pits

**FIGURE 5:**
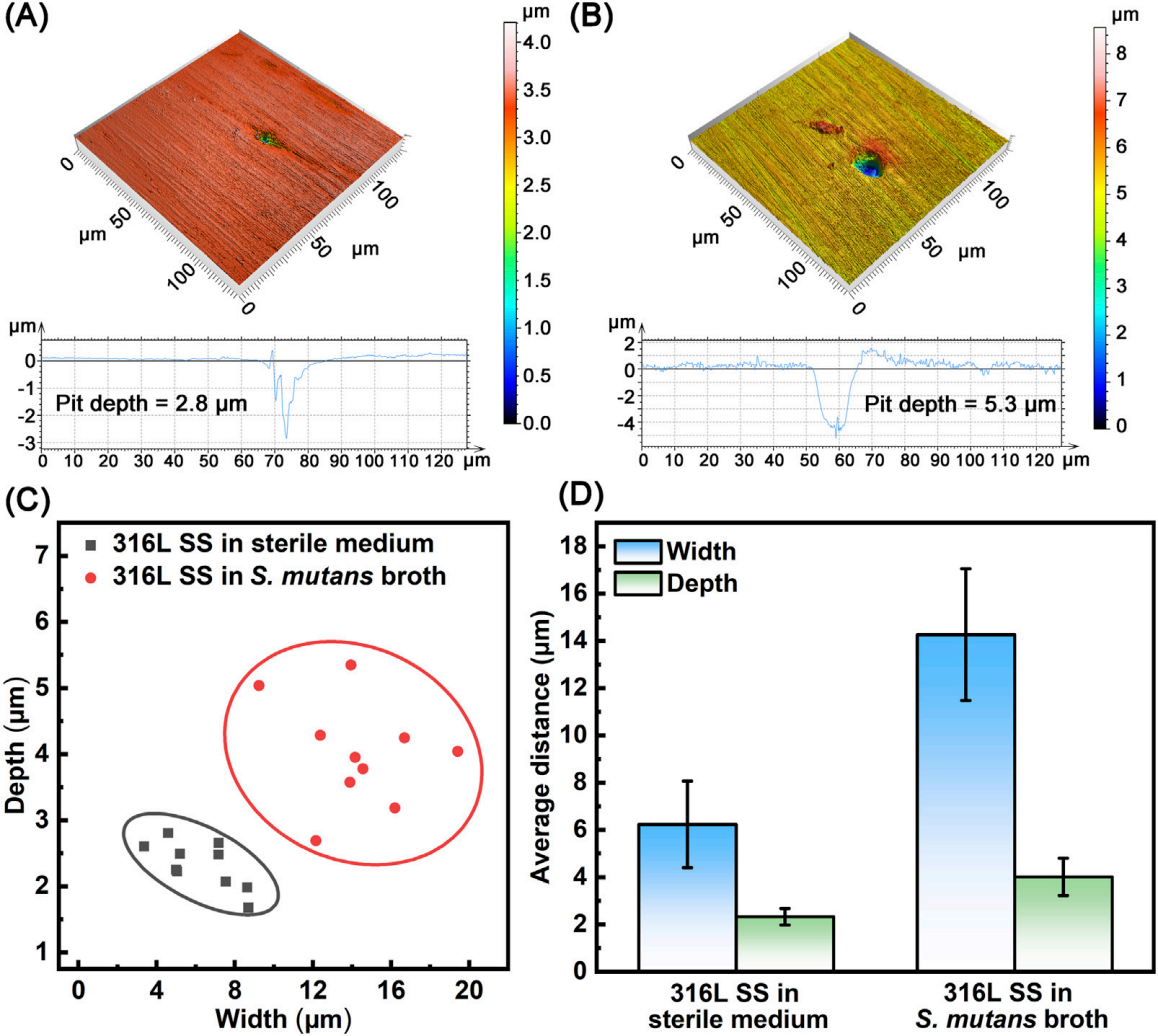
Analysis of surface corrosion morphology on 316L SS after 90 days of immersion in sterile and *S. mutans*-inoculated media: **(A)** 3D morphology of the deepest corrosion pit in sterile medium, **(B)** 3D morphology of the deepest corrosion pit immersed in *S. mutans* broth, **(C)** statistical analysis of corrosion pits, **(D)** average pit width and depth.

Localized corrosion, particularly pitting, is a major failure mode for biomedical materials in the oral environment (Rodrigues et al., 2013). To visually assess the long-term impact of microbial activity on surface integrity, after 90 days of immersion in either sterile or *S. mutans*-inoculated media, 3D surface morphology on 316L SS samples was analyzed by CLSM. Prior to analysis, biofilms and corrosion products were carefully removed from the surface. Figures 5A,B show the deepest corrosion pits observed on 316L SS surfaces under the two conditions. In the presence of *S. mutans*, typical circular pitting was observed, with a maximum pit depth of 5.3 μm–1.9 times deeper than that under sterile condition (2.8 μm). Figure 5C displays the statistical distribution of maximum pit depth and width on the sample surfaces, while Figure 5D summarizes the average maximum pit width and depth under these two conditions after 90 days of immersion. These results clearly indicated that the presence of *S. mutans* biofilms significantly accelerates pitting corrosion on 316L SS surfaces, as evidenced by the significant increase in both pit depth and width.

**FIGURE 6:**
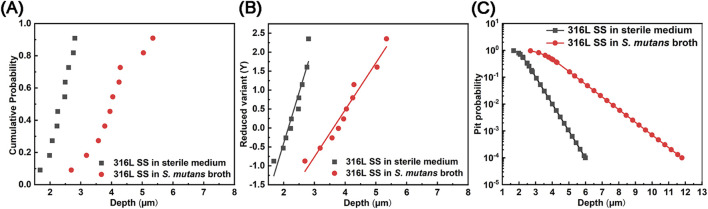
Statistical analysis of pitting corrosion probability on 316L SS after 90 days of immersion in sterile and *S. mutans*-inoculated media: **(A)** cumulative probability distribution, **(B)** Gumbel probability plot, and **(C)** probability distribution of pit depths.

The cumulative probability distribution of corrosion pit depths was statistically analyzed and is shown in Figure 6A. Compared to the sterile condition, the probability distribution of pit depths shifted toward deeper values in the *S. mutans*-inoculated medium. While pit depths in the sterile group were mainly in the range of 1.7–2.8 μm, those in the *S. mutans* group were concentrated between 2.7 and 5.3 μm. The Gumbel distribution function was further applied to analyze the corrosion events under both conditions (Figure 6B). The linear Gumbel distribution under both conditions suggests a statistically stable pit growth process, though localized metastable-to-stable transitions may still occur. The location parameter (*μ*), representing the most probable pit depth, and the scale parameter (*α*), reflecting the dispersion of pit depth values (Asadi and Melchers, 2017), were derived via linear fitting (Table 3). Both parameters increased markedly in the presence of *S. mutans*, indicating a notable enhancement in the severity and variability of pitting corrosion induced by microbial activity. Based on the Gumbel distribution, the predicted probability of pitting corrosion under each condition is presented in Figure 6C. The results show a markedly higher probability of pitting corrosion in the *S. mutans* group, further confirming that *S. mutans* significantly promotes the initiation and development of pitting corrosion on 316L SS under extended exposure periods. The increased depth and probability of pitting corrosion not only threaten the mechanical performance and service life of the oral materials but may also induce crack initiation, potentially resulting in unexpected failure. Furthermore, these pits act as preferential sites for the accumulation of aggressive ions, which locally accelerates corrosion and enhances the release of metal ions, thereby elevating the risk of inflammatory or allergic responses in the surrounding tissues.

**TABLE 3:** The location parameter (*μ*) and scale parameter (*α*) obtained via Gumbel probability linear fitting.

Experimental condition	Scale parameter (*α*)	Location parameter (*μ*)
316L SS in sterile medium	0.37	2.15
316L SS in *S. mutans* broth	0.81	3.61

### 3.4 pH variation during the long-term immersion

**FIGURE 7:**
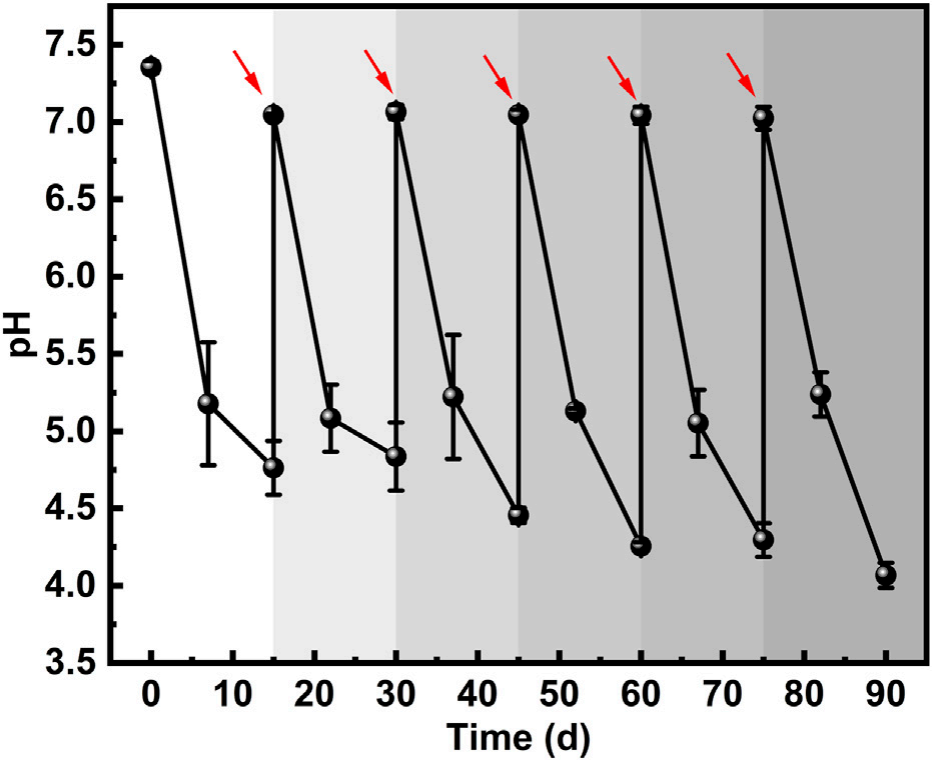
pH variation during the 90-day incubation of *S. mutans*-inoculated medium. Red arrows indicate the time points at which fresh medium was injected (on days 15, 30, 45, 60, and 75, respectively).

*S*. *mutans*, a typical acid-producing cariogenic bacterium, generates acidic metabolites that play a key role in metal corrosion (Yang Y. et al., 2021; Zhou et al., 2025). The pH of the *S. mutans*-inoculated medium was monitored periodically over the 90-day immersion period (Figure 7). At the beginning (day 0), the medium was adjusted to a neutral pH of 7.4. During each 15-day culture–replacement cycle, *S. mutans* continuously metabolized the sucrose in the medium via glycolysis, producing organic acids that led to a gradual decrease in pH. Due to a regular medium replacement, a clear oscillatory trend in pH was observed throughout the 90-day period. At the end of each cycle, the pH dropped to acidic values, reaching as low as 4.0–4.8. Moreover, the minimum pH values at the end of each cycle (days 15, 30, 45, 60, and 75) exhibited a gradual decrease over time, suggesting that *S. mutans* gradually adapted to the long-term culture conditions. By day 90, the pH of the medium had declined to as low as 4.0. It is important to note that the measured pH values represent the bulk solution conditions. Due to the diffusion barrier of the biofilm, the actual pH at the biofilm-metal interface is likely much lower. This localized acidic microenvironment critically contributes to the initiation of pitting corrosion.

### 3.5 Analysis of surface potential

**FIGURE 8:**
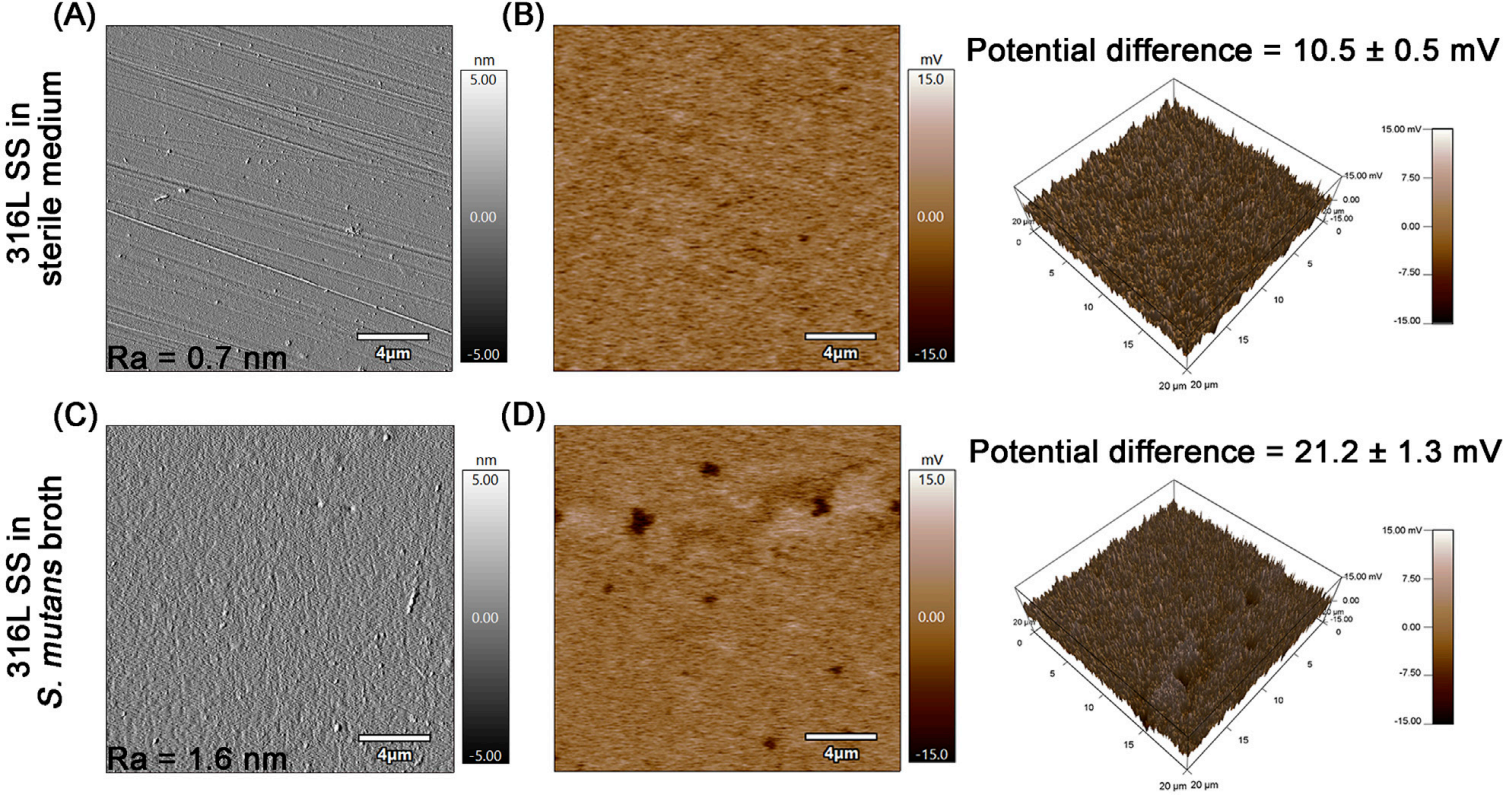
Surface morphology and surface potential distribution of 316L SS after 90-day immersion in sterile and *S. mutans*-inoculated media: **(A)** surface morphology in sterile medium, **(B)** surface potential map in sterile medium, **(C)** surface morphology in *S. mutans* broth, and **(D)** surface potential map in *S. mutans* broth.

To investigate the long-term effects of *S. mutans* on the integrity of the passive film, surface potential measurements were performed using the SKPFM mode of AFM after 90 days of immersion in both sterile and *S. mutans*-inoculated media (Figure 8). The samples used for SKPFM analysis required highly polished surfaces, resulting in significantly reduced roughness compared with those used in other experiments. Although the enhanced smoothness may have partially compromised the initial adhesion of *S. mutans*, pronounced changes in both corrosion morphology and surface potential distribution were still observed on the 316L SS surface after the 90-day prolonged exposure. The surface roughness (*R*_a_) of 316L SS increased from 0.7 nm in sterile medium to 1.6 nm in *S. mutans* broth. This result demonstrates that the surface of the specimen has undergone roughening, accompanied by the appearance of distinct corrosion characteristics (Chen et al., 2022). Alterations in surface roughness may further affect bacterial adhesion and metal corrosion processes. Roughened surfaces can promote microbial adhesion by providing additional anchorage sites, thereby accelerating biofilm maturation. This facilitates the formation of a microenvironment between the biofilm and the metal substrate, expediting the occurrence of localized corrosion. Furthermore, rough surfaces enhance the stability of the biofilm on the metal surface, enabling the persistent existence of the microenvironment and exerting a continuous impact on the metal. Under sterile conditions, the passive film on the surface of 316L SS remains relatively smooth and uniform, with a low surface potential difference of 10.5 ± 0.5 mV. In contrast, the surfaces of samples immersed in *S. mutans* culture broth exhibit significant heterogeneity, featuring scattered regions with notably lower potential (Figure 8B). The calculated surface potential difference increases to 21.2 ± 1.3 mV, approximately 2.02 times that under sterile conditions. A larger potential difference can lead to the formation of micro-galvanic cells, thereby enhancing the corrosion tendency (Narayanan et al., 2024). Regions rich in Cr/Mo elements exhibit higher cathodic properties; when exposed to corrosive media, more anodic regions deficient in Cr/Mo elements are preferentially attacked, resulting in selective dissolution (Narayanan et al., 2022; Narayanan et al., 2024). Under the action of *S. mutans*, passivating elements in the passive film may undergo dissolution, causing a local potential drop, accelerating the formation of galvanic cells, and further promoting the occurrence of localized corrosion.

### 3.6 Characterization of corrosion products

**FIGURE 9:**
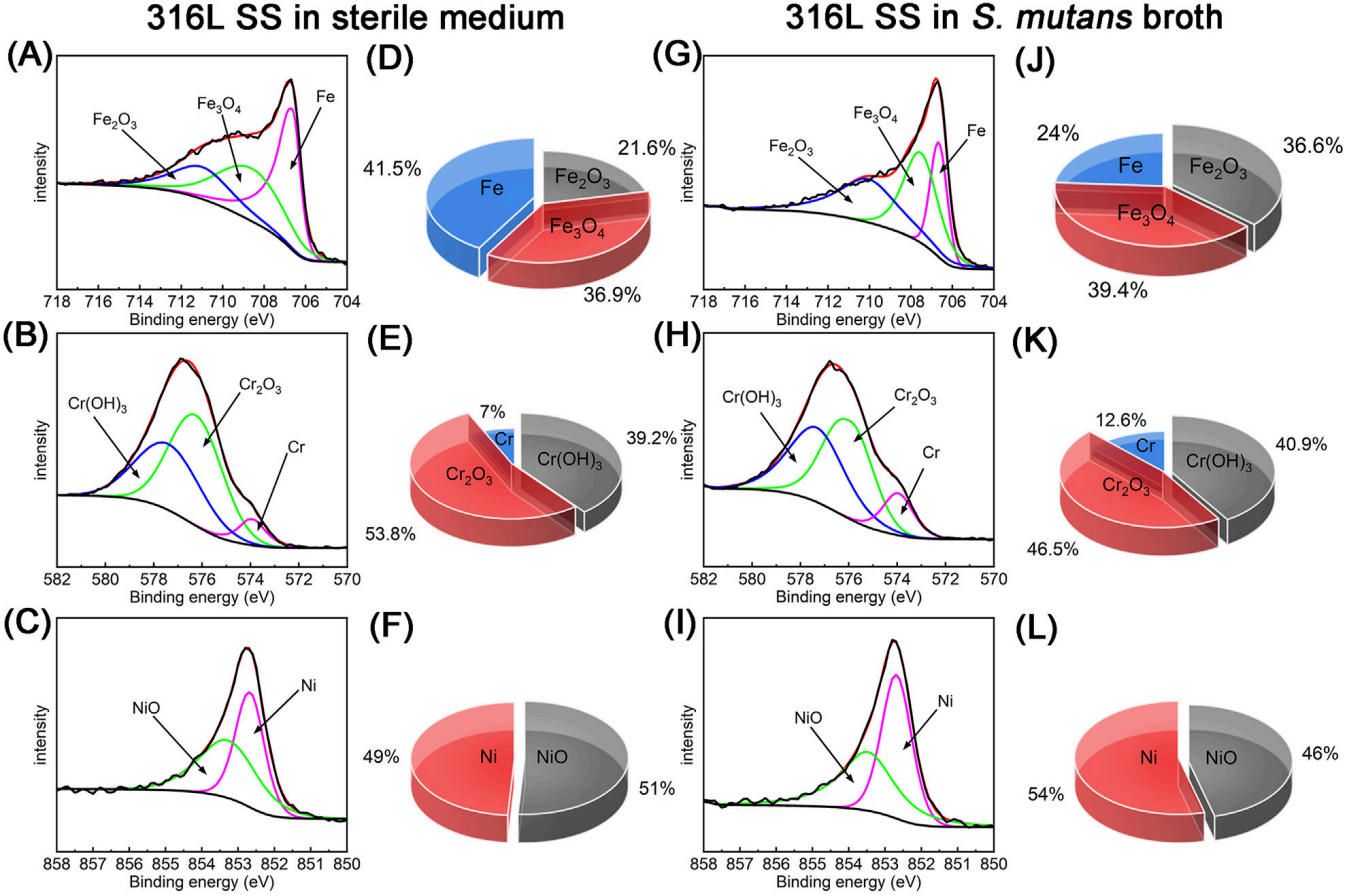
XPS analysis of corrosion products on 316L SS samples after 90 days of immersion in sterile and *S. mutans*-inoculated media: **(A–F)** show the high-resolution Fe 2p, Cr 2p, and Ni 2p spectra and corresponding relative content under sterile conditions; **(G–L)** present the Fe 2p, Cr 2p, and Ni 2p spectra and relative content under *S. mutans*-inoculated conditions.

After 90 days of immersion under different conditions, XPS was employed for semi-quantitative analysis of the corrosion products formed on 316L SS. Figure 9 displays the detailed chemical state of metal elements on the surfaces, along with the relative content of metal compounds. For samples immersed in sterile medium, XPS analysis revealed the presence of iron species in the outer layer of the passive film, with Fe_2_O_3_ accounting for 21.6%, Fe_3_O_4_ for 36.9%, and metallic Fe for 41.5%. Chromium was mainly present as Cr(OH)_3_ (39.2%) and Cr_2_O_3_ (53.8%), the latter, being abundant in the inner layer of the passive film, acts as a key contributor to corrosion resistance (Wang et al., 2020). Nickel existed as both NiO (51.0%) and metallic Ni (49.0%), and the presence of NiO was found to enhance the integrity and stability of the passive film (dos Santos Pereira et al., 2021). Notably, the ratio of chromium compounds to metallic chromium reached 13.29, indicating that chromium underwent preferential oxidation during passive film formation, resulting in a continuous and compact protective layer (Wang et al., 2019; Yu et al., 2024).

After 90 days of immersion in *S. mutans*-inoculated medium, the chemical composition of the 316L SS surface changed significantly. XPS analysis of the iron species revealed that the Fe_2_O_3_ content increased to 36.6%, Fe_3_O_4_ to 39.4%, while the proportion of metallic Fe decreased markedly to 24.0%. This shift might result from the dissolution of elemental iron due to microbial activity. High-resolution spectra of Cr showed a slight increase in Cr(OH)_3_–40.9%, while the proportion of Cr_2_O_3_, an oxide known for its excellent protective properties, decreased from 53.8% to 46.5%. This may lead to a defect within the passive film, compromising its corrosion resistance (Martinez et al., 2024). Similarly, the chemical state distribution of nickel changed noticeably, with NiO content decreasing from 51.0% to 46.0%. These findings suggest that acidic metabolic byproducts (e.g., lactic acid) and EPS produced by *S. mutans* adversely affected the chemical stability of the passive film on 316L SS, potentially compromising its corrosion resistance. This observation aligns well with electrochemical data (e.g., decreased *R*_p_ and increased *i*_corr_) and the more severe corrosion morphologies observed under microbial conditions.

## 4 Discussion

This study systematically investigated MIC behavior of *S*. *mutans* on the biomedical alloy 316L SS under simulated oral conditions over a 90-day period. The long-term continuous experiments demonstrated that *S. mutans* was capable of successfully colonizing the 316L SS surface and forming a progressively thickening and structurally complex biofilm. Electrochemical measurements, including OCP, LPR, EIS, and potentiodynamic polarization tests, along with analysis of corrosion morphology, consistently confirmed that the presence of *S. mutans* significantly accelerated the corrosion process of 316L SS during prolonged immersion. The periodic decrease in pH (dropping to as low as ∼4.0) due to *S. mutans*’ sustained acid production during each culture–replacement cycle further supported the ongoing metabolic activity and acidogenic potential of the bacteria. This acidification likely promoted the corrosion of 316L SS in the simulated oral environment. Analysis of corrosion products further revealed compositional degradation of the passive film. In the presence of *S. mutans*, protective chromium and nickel oxides were reduced, while iron dissolution intensified. Collectively, these findings provide substantial evidence that *S. mutans* exerts a significant MIC-accelerating effect on 316L SS through acidogenic metabolism under simulated long-term oral exposure.

Biofilms play a critical role during the MIC process of metallic materials (Qi et al., 2025). In this study, to ensure the long-term survival and metabolic activities of the microorganisms, the culture medium was replaced every 15 days. This operational process may have caused slight fluctuations in the solution, which could, in turn, influence the dynamic evolution of the biofilm. Nevertheless, such disturbances are unlikely to alter the overall maturation trend of the biofilm or the general corrosion progression occurring beneath it. During long-term service, microbial biofilms undergo complex structural and compositional evolution, which cannot be fully replicated by short-term studies (e.g., 14-day immersions). Such differences in biofilm development may lead to distinct corrosion behaviors. In previous short-term studies (e.g., 14 days) (Kaliaraj et al., 2018; Yang C. et al., 2021; Yang Y. et al., 2021), *S*. *mutans* was able to colonize the surface of 316L SS, but the resulting biofilm had not yet developed into a stable, compact structure. SEM and CLSM observations revealed that the biofilm at this stage was loosely structured and discontinuous, with large portions of the metal surface still exposed. Live/dead fluorescence staining showed a predominance of viable cells (green fluorescence), and the biofilm thickness was generally less than 40 μm (Yang Y. et al., 2021). Such an incomplete and discontinuous biofilm has limited ability to regulate the interfacial microenvironment. While organic acids produced by *S*. *mutans* may trigger corrosion, the loose biofilm offers little physical barrier or microenvironmental control, making the corrosion effect largely dependent on acidic metabolites. In contrast, the 90-day long-term study presented here captured the full evolution process of *S. mutans* biofilms on 316L SS. At day 3, scattered chain-like bacterial clusters were detected. By day 15, a continuous surface-covering biofilm was observed, and by day 90, the biofilm thickness increased to 88.1 ± 9.0 μm. CLSM combined with live/dead staining further showed a gradual increase in the proportion of non-viable cells over time. After day 15, red fluorescence becoming more uniformly distributed, which is likely associated with limited nutrient diffusion and intensified microbial competition within the mature biofilm. A well-developed and compact biofilm may influence metal corrosion through multiple mechanisms, including the formation of localized acidic microenvironments, interactions between EPS and metal ions, enzymatic activity, and physical shielding effects (Chang et al., 2024). These complex interactions always lead to more diverse and less predictable corrosion pathways (Qi et al., 2025).

Compared with previous short-term corrosion studies, the long-term tests in this work revealed significantly different corrosion morphologies and electrochemical behaviors. In short-term experiments, due to immature biofilm formation and underdeveloped acidic microenvironments, pitting corrosion was mostly in its early stages with shallow pits, typically up to 3.5 μm in depth (Yang C. et al., 2021). In contrast, under long-term exposure, the mature biofilm created a sustained acidic and physically restrictive environment, leading to more severe corrosion. After 90 days, the maximum pit depth ranged from 2.7 to 5.3 μm, and both the average maximum pit depth and width increased significantly. The Gumbel extreme value distribution further confirmed a higher probability of pitting events under long-term conditions, reflecting a substantial increase in corrosion risk driven by the persistent influence of the mature biofilm.

After 90-day immersion, the *i*_corr_ reached 6.4 ± 0.2 μA cm^−2^, which was higher than that observed after 7 days (about 5.6 μA cm^−2^) (Yang C. et al., 2021), indicating an increased corrosion tendency. Furthermore, this value is two orders of magnitude higher than that under sterile conditions, indicating that *S. mutans* seriously accelerates the corrosion of 316L SS. This acceleration poses a severe threat to the structural integrity, which may lead to unpredicted failure, shortened service life, and potential inflammatory responses. In addition, a distinct linear segment appeared in the low-frequency region of the Nyquist plot, attributed to diffusion limitations caused by the dense and compact biofilm. Consequently, a Warburg impedance element was introduced in the equivalent circuit fitting. At day 3, CLSM images revealed a discontinuous and loose biofilm structure. At this stage, the biofilm imposed minimal diffusion limitations, resulting in a negligible Warburg impedance. By day 15, the biofilm developed into a dense and compact architecture, accompanied by a notable increase in Warburg impedance of approximately three orders of magnitude, indicating significantly enhanced diffusion resistance. With prolonged incubation, the biofilm matured and increasing impeded mass transfer, leading to a continuous rise in Warburg impedance up to day 60. Between days 60 and 90, a slight decrease in Warburg impedance was observed, likely due to accumulation of dead microorganisms compromising biofilm integrity and reducing diffusion resistance. During short-term experiments, *S. mutans* formed discontinuous biofilms, leading to a limited impact of biofilms on diffusion. Thus, the influence of biofilm-mediated mass transport on metal corrosion is rarely addressed in previous studies (Yang Y. et al., 2021). These electrochemical characteristics highlight the dual role of mature biofilms during long-term immersion: promoting corrosion through sustained metabolic activity, while concurrently impeding mass transport via their compact physical structure.

The excellent corrosion resistance of 316L SS relies on its passive film, primarily composed of protective oxides such as Cr_2_O_3_ and NiO. However, under long-term exposure, the integrity of this film can be significantly compromised. AFM measurements revealed a notable increase in surface potential difference from 10.5 mV (sterile group) to 21.2 mV, accompanied by a widespread distribution of low-potential regions. This shift indicates a compromised surface passivation and a heightened susceptibility to corrosion (Narayanan et al., 2024). XPS analysis further confirmed a reduction in the content of key protective oxides: Cr_2_O_3_ decreased from 53.8% to 46.5%, and NiO from 51.0% to 46.0%, accompanied by a marked decline in elemental Fe content. These changes likely resulted from the acidic environment (with pH periodically dropping to ∼4.0) maintained by the mature biofilm over the 90-day period. More critically, once localized corrosion initiated, metal cation (e.g., Fe^2+^) release within pits facilitated the enrichment of aggressive ions such as Cl^−^, further accelerating the systematic breakdown of the passive film.

In contrast to short-term laboratory assays, long-term MIC process involves not only the cumulative effects of time but also the synergistic interplay of biofilm maturation, metabolic byproduct accumulation, and the persistent development of localized corrosion cells. Short-term studies predominantly capture the initial phase of MIC, emphasizing early bacterial colonization and metabolic activity. In contrast, long-term MIC involves the dynamic evolution of biofilm architecture and the physicochemical properties at the metal-solution interface. As biofilms mature from loosely organized to dense, robust structures, their capacity to modulate the local microenvironment is significantly enhanced. Concurrently, the sustained accumulation of metabolic byproducts, such as organic acids and EPS, fosters a persistently acidic milieu. Under prolonged microbial attack, the passive film may transition from localized degradation to widespread breakdown, markedly increasing both the depth and probability of pitting corrosion. These complex, time-dependent processes, which cannot be fully captured in short-term studies, more accurately reflect the real-world degradation of oral biomedical alloys during clinical service. Based on the findings of this study and previous research, oral microorganisms pose a persistent threat to the stability of medical alloys. Therefore, to ensure oral healthcare, the development and application of metallic materials or surface modification technologies with antimicrobial functionality can effectively mitigate MIC by inhibiting or disrupting biofilm adhesion and accumulation on material surfaces, thereby reducing the risk of corrosion-induced failure. Furthermore, for long-term indwelling oral medical devices, the use of alloys with superior corrosion resistance can minimize metal ion release and consequently prevent adverse biological responses such as peri-implant inflammation and allergic reactions.

In this study, we systematically investigated the acceleration effect and underlying mechanism of *S. mutans* on the corrosion of 316L SS under long-term exposure. It should be noted that the actual oral environment is far more complex. On one hand, physical factors such as variations in salivary flow and periodic temperature fluctuations can destabilize the biofilm structure formed on metallic surfaces. On the other hand, interactions among multiple coexisting microorganisms may produce synergistic effects with *S. mutans*, resulting in composite biofilms of greater structural and compositional complexity that further accelerate corrosion of 316L SS. In addition, other forms of corrosion also contribute to the failure of metallic materials in the oral environment. Among them, crevice corrosion represents a typical failure mode, commonly occurring at implant–abutment interfaces and bracket–archwire gaps. These confined regions generate localized microenvironments that differ markedly from the bulk solution, thereby amplifying the localized corrosion of metals.

## 5 Conclusion

In this study, a long-term anaerobic investigation of the MIC behavior of 316L SS induced by *S*. *mutans* was performed. *S. mutans* was found to form a progressively thickened biofilm, transitioning from an initially sparse structure to a dense and compact matrix, reaching a thickness of 88 μm by day 90. Electrochemical measurements revealed a pronounced corrosion acceleration in the presence of *S. mutans*. Compared to the sterile condition, the *E*_OCP_ exhibited a sharp decline, the *R*_p_ dropped to 20.9 ± 6.6 kΩ cm^2^, the *i*_corr_ increased by two orders of magnitude at the end of 90-day immersion. These findings confirm the significant corrosion-promoting effect of *S. mutans* over prolonged periods. Surface analyses revealed that *S. mutans* markedly aggravated pitting corrosion, with the maximum pit depth reaching 5.3 μm and the average maximum pit width and depth increasing significantly. Periodic pH measurements of the *S. mutans*-inoculated broth showed cyclical acidification during medium replacement intervals, with pH values dropping to approximately 4.0 by day 90, indicating persistent acid production. This sustained acidic environment compromised the stability of the passive film, as evidenced by AFM surface potential mapping, which showed a more than twofold increase in surface potential variation and widespread low-potential regions compared to the sterile control. XPS analysis further revealed a marked decline in the protective Cr_2_O_3_ and NiO species within the passive film, along with a reduction in elemental Fe content, implying degradation of the corrosion-resistant barrier and increased iron dissolution. Compared to short-term MIC studies, 90-day immersion resulted in significantly more severe corrosion damage. Collectively, these results provide mechanistic insights into the long-term corrosion behavior of oral biomedical alloys under microbial challenge and highlight the necessity of developing targeted MIC mitigation strategies in dental materials.

## Data Availability

The raw data supporting the conclusions of this article will be made available by the authors, without undue reservation.

## References

[B1] Alam M. K. Zheng L. Liu R. Papagerakis S. Papagerakis P. Geyer C. R. (2018). Synthetic antigen-binding fragments (fabs) against *S. mutans* and *S. sobrinus* inhibit caries formation. Sci. Rep. 8 (1), 10173. 10.1038/s41598-018-28240-0 29976956 PMC6033933

[B2] Asadi Z. S. Melchers R. E. (2017). Extreme value statistics for pitting corrosion of old underground cast iron pipes. Reliab. Eng. and Syst. Saf. 162, 64–71. 10.1016/j.ress.2017.01.019

[B3] Asri R. I. M. Harun W. S. W. Samykano M. Lah N. A. C. Ghani S. A. C. Tarlochan F. (2017). Corrosion and surface modification on biocompatible metals: a review. Mater Sci. Eng. C Mater Biol. Appl. 77, 1261–1274. 10.1016/j.msec.2017.04.102 28532004

[B4] Beech I. B. Sunner J. (2004). Biocorrosion: towards understanding interactions between biofilms and metals. Curr. Opin. Biotechnol. 15 (3), 181–186. 10.1016/j.copbio.2004.05.001 15193324

[B5] Blackwood D. J. (2018). An electrochemist perspective of microbiologically influenced corrosion. Corros. Mater. Degrad. 1 (1), 59–76. 10.3390/cmd1010005

[B6] Bowen W. H. Koo H. (2011). Biology of *Streptococcus mutans*-derived glucosyltransferases: role in extracellular matrix formation of cariogenic biofilms. Caries Res. 45 (1), 69–86. 10.1159/000324598 21346355 PMC3068567

[B7] Chang W. Qian H. Li Z. Mol A. Zhang D. (2024). Application and prospect of localized electrochemical techniques for microbiologically influenced corrosion: a review. Corros. Sci. 236, 112246. 10.1016/j.corsci.2024.112246

[B8] Chen X. Xiao C. Wang X. Yang J. He C. (2022). Corrosion behaviors of 2205 duplex stainless steel in biotic and abiotic NaCl solutions. Constr. Build. Mater. 342, 127699. 10.1016/j.conbuildmat.2022.127699

[B9] Chiniforoush E. A. Gholizadeh T. Jandaghi M. R. Moverare J. Gür C. H. (2025). Impact of active to inert shielding gas transition on the corrosion behavior of wire arc additively manufactured duplex stainless steel. Mater. and Des. 253, 113907. 10.1016/j.matdes.2025.113907

[B10] dos Santos Pereira R. Droppa R. Lopes de Oliveira M. C. Antunes R. A. (2021). Effect of milling parameters on the stability of the passive film of AISI 304 stainless steel. J. Mater. Eng. Perform. 30 (11), 8131–8144. 10.1007/s11665-021-06064-w

[B11] Dzidic M. Collado M. C. Abrahamsson T. Artacho A. Stensson M. Jenmalm M. C. (2018). Oral microbiome development during childhood: an ecological succession influenced by postnatal factors and associated with tooth decay. ISME J. 12 (9), 2292–2306. 10.1038/s41396-018-0204-z 29899505 PMC6092374

[B12] Eduok U. (2024). Microbiologically induced intergranular corrosion of 316L stainless steel dental material in saliva. Mater. Chem. Phys. 313, 128799. 10.1016/j.matchemphys.2023.128799

[B13] Fulaz S. Hiebner D. Barros C. H. N. Devlin H. Vitale S. Quinn L. (2019). Ratiometric imaging of the *in situ* pH distribution of biofilms by use of fluorescent mesoporous silica nanosensors. ACS Appl. Mater. and Interfaces 11 (36), 32679–32688. 10.1021/acsami.9b09978 31418546

[B14] Gopalakrishnan U. Felicita A. S. Mahendra L. Kanji M. A. Varadarajan S. Raj A. T. (2021). Assessing the potential association between microbes and corrosion of intra-oral metallic alloy-based dental appliances through a systematic review of the literature. Front. Bioeng. Biotechnol. 9, 631103. 10.3389/fbioe.2021.631103 33791285 PMC8005604

[B15] Gu T. Jia R. Unsal T. Xu D. (2019). Toward a better understanding of microbiologically influenced corrosion caused by sulfate reducing bacteria. J. Mater. Sci. and Technol. 35 (4), 631–636. 10.1016/j.jmst.2018.10.026

[B16] Hayes A. Sharifi S. Stack M. M. (2015). Micro-abrasion-corrosion maps of 316L stainless steel in artificial saliva. J. Bio- Tribo-Corrosion 1 (2), 15. 10.1007/s40735-015-0015-y

[B17] Hollmann B. Perkins M. Chauhan V. M. Aylott J. W. Hardie K. R. (2021). Fluorescent nanosensors reveal dynamic pH gradients during biofilm formation. NPJ Biofilms Microbiomes 7 (1), 50. 10.1038/s41522-021-00221-8 34140515 PMC8211749

[B18] Hong Q. Huo S. Tang H. Qu X. Yue B. (2021). Smart nanomaterials for treatment of biofilm in orthopedic implants. Front. Bioeng. Biotechnol. 9, 694635. 10.3389/fbioe.2021.694635 34589470 PMC8473796

[B19] Jia R. Unsal T. Xu D. Lekbach Y. Gu T. (2019). Microbiologically influenced corrosion and current mitigation strategies: a state of the art review. Int. Biodeterior. and Biodegrad. 137, 42–58. 10.1016/j.ibiod.2018.11.007

[B20] Jin Y. Li J. Zhang M. Zheng B. Xu D. Gu T. (2024). Effect of exogenous flavins on the microbial corrosion by geobacter sulfurreducens *via* iron-to-microbe electron transfer. J. Mater. Sci. and Technol. 171, 129–138. 10.1016/j.jmst.2023.06.014

[B21] Kaliaraj G. S. Vishwakarma V. Kirubaharan K. Dharini T. Ramachandran D. Muthaiah B. (2018). Corrosion and biocompatibility behaviour of zirconia coating by EBPVD for biomedical applications. Surf. Coatings Technol. 334, 336–343. 10.1016/j.surfcoat.2017.11.047

[B22] Kameda T. Oda H. Ohkuma K. Sano N. Batbayar N. Terashima Y. (2014). Microbiologically influenced corrosion of orthodontic metallic appliances. Dent. Mater J. 33 (2), 187–195. 10.4012/dmj.2013-297 24583645

[B23] Kaur G. Rajesh S. Princy S. A. (2015). Plausible drug targets in the *Streptococcus mutans* quorum sensing pathways to combat dental biofilms and associated risks. Indian J. Microbiol. 55 (4), 349–356. 10.1007/s12088-015-0534-8 26543259 PMC4627952

[B24] Lee Y. H. Chung S. W. Auh Q. S. Hong S. J. Lee Y. A. Jung J. (2021). Progress in oral microbiome related to oral and systemic diseases: an update. Diagn. (Basel) 11 (7), 1283. 10.3390/diagnostics11071283 34359364 PMC8306157

[B25] Lemos J. A. Palmer S. R. Zeng L. Wen Z. T. Kajfasz J. K. Freires I. A. (2019). The biology of *Streptococcus mutans* . Microbiol. Spectr. 7 (1), 7.1.03. 10.1128/microbiolspec.GPP3-0051-2018 30657107 PMC6615571

[B26] Li Y. Ning C. (2019). Latest research progress of marine microbiological corrosion and bio-fouling, and new approaches of marine anti-corrosion and anti-fouling. Bioact. Mater 4, 189–195. 10.1016/j.bioactmat.2019.04.003 31192994 PMC6513773

[B27] Li H. Wang Y. Zhao X. Yan Z. Song C. Wang S. (2023). Chirality of tyrosine controls biofilm formation *via* the regulation of bacterial adhesion. Biochem. Eng. J. 192, 108844. 10.1016/j.bej.2023.108844

[B28] Li Z. Sun M. Xia Q. Yu Z. Zhao M. Li W. (2024). Accelerated microbial corrosion of 316 L SS in extreme acidic environment by a typical bioleaching strain Acidithiobacillus ferrooxidans. Corros. Sci. 238, 112353. 10.1016/j.corsci.2024.112353

[B29] Liu X. Zhou E. Fan Y. Wang F. Xu D. (2025). Riboflavin-mediated extracellular electron transfer enhances microbiologically influenced corrosion of 316L stainless steel by enterococcus faecalis. Bioelectrochemistry 165, 108982. 10.1016/j.bioelechem.2025.108982 40209334

[B30] Lodhi M. J. K. Deen K. M. Greenlee-Wacker M. C. Haider W. (2019). Additively manufactured 316L stainless steel with improved corrosion resistance and biological response for biomedical applications. Addit. Manuf. 27, 8–19. 10.1016/j.addma.2019.02.005

[B31] Madiba M. Oluremi B. B. Gulube Z. Oderinlo O. O. Marimani M. Osamudiamen P. M. (2023). Anti-*Streptococcus mutans*, anti-adherence and anti-acidogenic activity of uvaria chamae P. Beauv. J. Ethnopharmacol. 300, 115673. 10.1016/j.jep.2022.115673 36096348

[B32] Madirisha M. Hack R. van der Meer F. (2022). Simulated microbial corrosion in oil, gas and non-volcanic geothermal energy installations: the role of biofilm on pipeline corrosion. Energy Rep. 8, 2964–2975. 10.1016/j.egyr.2022.01.221

[B33] Manohar A. K. Bretschger O. Nealson K. H. Mansfeld F. (2008). The use of electrochemical impedance spectroscopy (EIS) in the evaluation of the electrochemical properties of a microbial fuel cell. Bioelectrochemistry 72 (2), 149–154. 10.1016/j.bioelechem.2008.01.004 18294928

[B34] Martinez A. Narayanan D. Case R. Castaneda H. Radwan A. B. Bhadra J. (2024). Pit initiation mechanism of modified martensitic 13Cr stainless steel exposed to CO2 saturated acidic environments at elevated temperatures induced by Ti(C,N) inclusions. Electrochimica Acta 475, 143655. 10.1016/j.electacta.2023.143655

[B35] Moradi M. Ghiara G. Spotorno R. Xu D. Cristiani P. (2022). Understanding biofilm impact on electrochemical impedance spectroscopy analyses in microbial corrosion and microbial corrosion inhibition phenomena. Electrochimica Acta 426, 140803. 10.1016/j.electacta.2022.140803

[B36] Narayanan D. Liu M. Kuttolamadom M. Castaneda H. (2022). Identification and development of a new local corrosion mechanism in a Laser Engineered Net Shaped (LENS) biomedical co-cr-mo alloy in Hank’s solution. Corros. Sci. 207, 110599. 10.1016/j.corsci.2022.110599

[B37] Narayanan D. Martinez A. Martin U. Mansoor B. Case R. Castaneda H. (2024). Localized corrosion in selective laser melted SS316L in CO(2) and H(2)S brines at elevated temperatures. Npj Mater Degrad. 8 (1), 50. 10.1038/s41529-024-00468-4 38736645 PMC11087244

[B38] Nishikata A. Suzuki F. Tsuru T. (2005). Corrosion monitoring of nickel-containing steels in marine atmospheric environment. Corros. Sci. 47 (10), 2578–2588. 10.1016/j.corsci.2004.10.009

[B39] Oh K. T. Kim Y. S. Park Y. S. Kim K. N. (2004). Properties of super stainless steels for orthodontic applications. J. Biomed. Mater Res. B Appl. Biomater. 69 (2), 183–194. 10.1002/jbm.b.30002 15116408

[B40] Olmedo D. G. Nalli G. Verdu S. Paparella M. L. Cabrini R. L. (2013). Exfoliative cytology and titanium dental implants: a pilot study. J. Periodontol. 84 (1), 78–83. 10.1902/jop.2012.110757 22414261

[B41] Perera D. McLean A. Morillo-Lopez V. Cloutier-Leblanc K. Almeida E. Cabana K. (2022). Mechanisms underlying interactions between two abundant oral commensal bacteria. ISME J. 16 (4), 948–957. 10.1038/s41396-021-01141-3 34732850 PMC8940909

[B42] Procopio L. (2019). The role of biofilms in the corrosion of steel in marine environments. World J. Microbiol. Biotechnol. 35 (5), 73. 10.1007/s11274-019-2647-4 31037431

[B43] Qi P. Zeng Y. Zhang D. Sun Y. Wang P. (2025). The biofilm-metal interface: a hotspot for microbiologically influenced corrosion. Cell Rep. Phys. Sci. 6 (3), 102500. 10.1016/j.xcrp.2025.102500

[B44] Rao P. Mulky L. (2023). An overview of microbiologically influenced corrosion on stainless steel. ChemBioEng Rev. 10 (5), 829–840. 10.1002/cben.202300001

[B45] Rodrigues D. C. Valderrama P. Wilson T. G. Palmer K. Thomas A. Sridhar S. (2013). Titanium corrosion mechanisms in the oral environment: a retrieval study. Mater. (Basel) 6 (11), 5258–5274. 10.3390/ma6115258 28788388 PMC5452779

[B46] Sheit H. M. K. Mohan K. S. Srinivasan P. Muthu S. E. Dinesh A. Rajeswari B. (2024). Anti-corrosive efficiency of salvadora persica plant stick powder on SS 316L orthodontic wire in artificial saliva. Results Chem. 12, 101894. 10.1016/j.rechem.2024.101894

[B47] Tang Y. C. Katsuma S. Fujimoto S. Hiromoto S. (2006). Electrochemical study of type 304 and 316L stainless steels in simulated body fluids and cell cultures. Acta Biomater. 2 (6), 709–715. 10.1016/j.actbio.2006.06.003 16935040

[B48] Tuck B. Leinecker N. Watkin E. Somers A. Forsyth M. Machuca L. L. (2022). Efficiency of a novel multifunctional corrosion inhibitor against biofilms developed on carbon steel. Front. Bioeng. Biotechnol. 10, 803559. 10.3389/fbioe.2022.803559 35127661 PMC8814422

[B49] Wang Z. Paschalidou E.-M. Seyeux A. Zanna S. Maurice V. Marcus P. (2019). Mechanisms of Cr and Mo enrichments in the passive oxide film on 316L austenitic stainless steel. Front. Mater. 6, 232. 10.3389/fmats.2019.00232

[B50] Wang L. Seyeux A. Marcus P. (2020). Thermal stability of the passive film formed on 316L stainless steel surface studied by ToF-SIMS. Corros. Sci. 165, 108395. 10.1016/j.corsci.2019.108395

[B51] Wang Q. Zhang M. Yang C. Yang Y. Zhou E. Liu P. (2022). Oral microbiota accelerates corrosion of 316L stainless steel for orthodontic applications. J. Mater. Sci. and Technol. 128, 118–132. 10.1016/j.jmst.2022.04.012

[B52] Wong A. Subar P. E. Young D. A. (2017). Dental caries: an update on dental trends and therapy. Adv. Pediatr. 64 (1), 307–330. 10.1016/j.yapd.2017.03.011 28688595

[B53] Xu W. Yu F. Yang L. Zhang B. Hou B. Li Y. (2018). Accelerated corrosion of 316L stainless steel in simulated body fluids in the presence of H(2)O(2) and albumin. Mater Sci. Eng. C Mater Biol. Appl. 92, 11–19. 10.1016/j.msec.2018.06.023 30184732

[B54] Xu D. Gu T. Lovley D. R. (2023). Microbially mediated metal corrosion. Nat. Rev. Microbiol. 21 (11), 705–718. 10.1038/s41579-023-00920-3 37344552

[B55] Xu W. Yu F. Addison O. Zhang B. Guan F. Zhang R. (2024). Microbial corrosion of metallic biomaterials in the oral environment. Acta Biomater. 184, 22–36. 10.1016/j.actbio.2024.06.032 38942189

[B56] Yang C. Wang Q. Ren Y. Jin D. Liu D. Moradi M. (2021a). Corrosion behavior of high nitrogen nickel-free austenitic stainless steel in the presence of artificial saliva and *Streptococcus mutans* . Bioelectrochemistry 142, 107940. 10.1016/j.bioelechem.2021.107940 34492448

[B57] Yang Y. Masoumeh M. Zhou E. Liu D. Song Y. Xu D. (2021b). *Streptococcus mutans* biofilms induce metabolite-mediated corrosion of 316 L stainless steel in a simulated oral environment. Corros. Sci. 182, 109286. 10.1016/j.corsci.2021.109286

[B58] Yu K. P. Jiang H. Xu X. Y. Huang M. X. (2024). Design of corrosion-resistant alloys for preventing oxidation-induced nanoscale Cr-depletion by inclusion engineering. Mater. and Des. 244, 113146. 10.1016/j.matdes.2024.113146

[B59] Zheng Y. Yang Y. Liu X. Liu P. Li X. Zhang M. (2024). Accelerated corrosion of 316L stainless steel in a simulated oral environment *via* extracellular electron transfer and acid metabolites of subgingival microbiota. Bioact. Mater 35, 56–66. 10.1016/j.bioactmat.2024.01.007 38283387 PMC10810744

[B60] Zhou X. Zhang Q. Lu J. Zheng Y. Wu L. Xu D. (2023). Enhanced corrosion resistance and biofilm inhibition of functionally graded TC4/TC4-5Cu fabricated by selective laser melting against *Streptococcus mutans* . Acta Metall. Sin. Engl. Lett. 36 (12), 1961–1978. 10.1007/s40195-023-01622-8

[B61] Zhou X. Leng A. Li C. Tang T. Xu J. Wei B. (2025). The role of oral microbiota in accelerating corrosion of Ti6Al4V: an electrochemical study. Mater. Chem. Phys. 340, 130836. 10.1016/j.matchemphys.2025.130836

[B62] Zou J. Meng M. Law C. S. Rao Y. Zhou X. (2018). Common dental diseases in children and malocclusion. Int. J. Oral Sci. 10 (1), 7. 10.1038/s41368-018-0012-3 29540669 PMC5944594

